# Evaluation of somatostatin and nucleolin receptors for therapeutic delivery in non-small cell lung cancer stem cells applying the somatostatin-analog DOTATATE and the nucleolin-targeting aptamer AS1411

**DOI:** 10.1371/journal.pone.0178286

**Published:** 2017-05-22

**Authors:** Sif Holmboe, Pernille Lund Hansen, Helge Thisgaard, Ines Block, Carolin Müller, Niels Langkjær, Poul Flemming Høilund-Carlsen, Birgitte Brinkmann Olsen, Jan Mollenhauer

**Affiliations:** 1 Lundbeckfonden Center of Excellence NanoCAN, University of Southern Denmark, Odense, Denmark; 2 Molecular Oncology, University of Southern Denmark, Odense, Denmark; 3 Department of Nuclear Medicine, Odense University Hospital, Odense, Denmark; 4 Department of Clinical Research, University of Southern Denmark, Odense, Denmark; Università degli Studi della Campania "Luigi Vanvitelli", ITALY

## Abstract

Cancer stem cells represent the putative tumor-driving subpopulation thought to account for drug resistance, relapse, and metastatic spread of epithelial and other cancer types. Accordingly, cell surface markers for therapeutic delivery to cancer stem cells are subject of intense research. Somatostatin receptor 2 and nucleolin are known to be overexpressed by various cancer types, which have elicited comprehensive efforts to explore their therapeutic utilization. Here, we evaluated somatostatin receptor 2 targeting and nucleolin targeting for therapeutic delivery to cancer stem cells from lung cancer. Nucleolin is expressed highly but not selectively, while somatostatin receptor 2 is expressed selectively but not highly by cancer cells. The non-small cell lung cancer cell lines A549 and H1299, displayed average levels of both surface molecules as judged based on analysis of a larger cell line panel. H1299 compared to A549 cells showed significantly elevated sphere-forming capacity, indicating higher cancer stem cell content, thus qualifying as suitable test system. Nucleolin-targeting ^57^Co-DOTA-AS1411 aptamer showed efficient internalization by cancer cells and, remarkably, at even higher efficiency by cancer stem cells. In contrast, somatostatin receptor 2 expression levels were not sufficiently high in H1299 cells to confer efficient uptake by either non-cancer stem cells or cancer stem cells. The data provides indication that the nucleolin-targeting AS1411 aptamer might be used for therapeutic delivery to non-small cell lung cancer stem cells.

## Introduction

Lung cancer is the most common cause of cancer death in industrialized countries, with non-small cell lung cancer (NSCLC) as the most common form accounting for about 80% of the cases [1, 2]. NSCLC is often diagnosed after the occurrence of metastases. At this stage, a curative therapy is no longer an option and a rapid disease progression results in five year survival rates below 15% [2].

Cancer stem cells (CSCs) represent a small subpopulation of the cancer cells with stem-like properties such as ability for self-renewal and asymmetric division, that enables them to restore heterogeneous tumors [3–5]. After their initial discovery in breast cancer, CSCs were subsequently also found in various other solid cancer types, including NSCLC [4–7]. Of importance, CSCs display high tumorigenicity, elevated drug resistance and propensity for metastatic spread, and therefore are thought to be responsible for relapse of drug resistant metastatic cancer [4–7]. This has elicited intense searches for biomarkers for, and therapeutic strategies against CSCs in general, and NSCLC-CSCs in particular. Different cell surface proteins are presently discussed to identify NSCLC-CSCs including, CD133, EpCAM, CXCR4, and ABCG2 [6, 7]. A common property across cancer types is the ability to form tumor spheres under non-adherent culture conditions, in the presence of defined growth factors. This has advanced to a standard assay for determining the CSC numbers [5–7].

Small molecule drugs including chemotherapeutics, have the advantage of rapid uptake by cancer cells, but the disadvantage of rapid extrusion by CSCs, via multiple drug resistance proteins, such as ABCG2 [6, 7]. Macromolecular drugs, such as nucleic acids mediating RNA-interference, would have the advantage to escape these extrusion mechanisms [8–11].

However, these drugs are typically entering cells at low efficiency, thus requiring special delivery mechanisms [8–11]. Somatostatin receptor 2 (SSTR2) and nucleolin (NCL) are under intense investigation, based on their overexpression at the surface of cancer cells [12–18]. SSTR2 is a cell surface receptor overexpressed in neuroendocrine tumors [12–14] and peptide-based SSTR2-targeting, for example by radiolabeled DOTATATE, is already used for diagnostic imaging. Furthermore, the potential of DOTATATE for delivery of therapeutic agents has been explored in various studies [12–14, 18]. While SSTR2 is a classical cell surface receptor, NCL was discovered by chance, tracing back to the identification of a G-quadruplex forming aptamer, later on referred to as AS1411, with anti-cancer activity [15–17, 19]. NCL commonly locates to the nucleus, but AS1411 was shown to bind to NCL at the surface of cancer cells, where the protein is also located for yet unclear reasons [17]. Development of AS1411 reached clinical phase trial 2 in renal cancer, where, however, it failed to show efficacy [19]. Investigations are presently ongoing to evaluate, whether AS1411 can be used for drug delivery, including proof-of-concept that the aptamer may qualify for the delivery of nucleic acid-based therapeutics to cancer cells [10, 11].

Here, we set out to explore, whether SSTR2 or NCL can be utilized for efficient delivery of radionuclides to NSCLC-CSCs. We established H1299-derived spheres as CSC model system. We report that, although NCL does not display strong tendency towards selective overexpression by cancer cells, efficient uptake into NSCLC-CSCs was conferred by the NCL-targeting AS1411 aptamer. This is in contrast to SSTR2, which was more selectively overexpressed by cancer cells, but SSTR2-targeting DOTATATE was inferior for delivery purposes, as can be explained by low expression of the receptor.

## Materials and methods

### Cell culture

Cell lines were grown in a humidified incubator (at 37°C and 5% CO_2_). For passaging, cells were washed with phosphate buffered saline, and detached with TrypLE Express (Invitrogen, Karlsruhe, Germany). A549 (ATCC, CLL-185) was grown in Dulbecco’s Modified Eagle’s Medium (Sigma-Aldrich, Copenhagen, Denmark), and H1299 (ATCC, CLR-5803) was grown in Roswell Park Memorial Institute 1640 medium with L-glutamine (Sigma-Aldrich). Both media were supplemented with 10% fetal bovine serum (Sigma-Aldrich), and 1% Penicillin-Streptomycin (Sigma-Aldrich). All experiments were performed with 80% confluent cells. All pictures of cells were taken using the IX71^®^ Inverted Microscope (Olympus Ballerup, Denmark), with a 10X/0.3phC lens.

In order to create the comparative RNA and protein panels, the following cell lines were used; 184A1 (CRL-8798), MCF-10A (CRL-10317), MCF-12A (CRL-10782), ZR-75-1 (CRL-1500), MCF7 (HTB-22), T47-D (HTB-133), MDA-MB-361 (HTB-27), BT-474 (HTB-20), HCC70 (CRL-2315), BT-20 (HTB-19), MDA-MB-231 (CRM-HTB-26), HCC1500 (CRL-2329), HCC1569 (CRL-2330), BT-549 (HTB-122), MDA-MB-436 (HBT-130), NCI-H69 (HTB-119), PC-3 (CRL-1435) and U-251 MG (formerly known as U-373 MG) (ECACC 09063001). All were obtained from ATCC (Wesel, Germany) except for U-251 MG that was obtained at the ECACC (Porton Down, UK). Cell lines were maintained following the guidelines of the providers.

### Quantitative RT-PCR

Total RNA was harvested from adherent cells using the RNeasy Mini Kit (Qiagen, Copenhagen, Denmark) according to the instructions of the supplier. The RNA concentration was measured on the NanoDrop ND-1000 spectrophotometer (Thermo Fisher Scientific, Hvidovre, Denmark), and stored at -80°C until use. RNA was reverse transcribed using the RevertAid Minus First strand cDNA synthesis kit (Life Technologies, Nærum, Denmark) using 1 μg total RNA and oligo(dT) primers, following the instructions of the manufacturer. qRT-PCR was performed with Maxima^®^ Probe/ROX qPCR Master Mix (Thermo Fisher Scientific) and taqman assays for *NCL* (Life Technologies, Hs01066668-m1) and *SSTR2* (Life Technologies, Hs00265624_s1). 10 ng cDNA was used per reaction, as recommended by the providers’ protocols. *ACTB* (Life Technologies, Hs99999903-m1) and *GAPDH* (Life Technologies, Hs99999905-m1) were used as references for normalization. The qRT-PCR was performed in a ABI Prism 7300 (Thermo Fisher Scientific), for 2 min at 50°C, followed by 15 min at 95°C and 40 cycles at 95°C for 15 sec and 60°C for 1 min. Expression levels were analyzed using the Biogazelle qBase^plus^ software (www.qbaseplus.com), and normalized to *ACTB* and *GAPDH*. Moreover, the expression levels were referred to a virtual common reference (REF), representing the mean expression level of the *TP53* and the *CDKN1A* mRNA in MCF7 cells.

### Western blot analysis

Adherent cells were harvested in proteolytic RIPA buffer, mixed with EDTA-Free protease inhibitor (Roche, Hvidovre, Denmark). The protein concentration was measured by the Pierce^™^ BCA protein assay kit (Thermo Fisher Scientific). Gel electrophoresis was performed under reducing conditions with 10% SDS-polyacrylamide gels. Proteins were blotted onto a polyvinylidene difluoride (PVDF) membrane and the membrane incubated with a monoclonal mouse anti-NCL IgG1 antibody (1:5000, 4E2, Abcam, Cambridge, UK) [20]. After rinsing, the membrane was incubated with HRP conjugated polyclonal rabbit-anti-mouse secondary antibody (1:5000, Jackson Immunoresearch, Suffolk, UK) and visualized on X-ray film using Super-Signal West Dura Extended Duration substrate kit according to the manufacturer’s instructions (Thermo Fisher Scientific).

After stripping, the process was repeated on the same membrane with a monoclonal mouse GAPDH-specific IgM antibody (1:5000, 3C2, Sigma-Aldrich). Developed films were digitalized, and the protein expression levels were quantified with the Image Studio^™^ Litesoftware (www.licor.com). NCL expression levels were adjusted to the individual GAPDH levels and, to compare, normalized to the mean value of the normal epithelial breast cell lines 184A1 and MCF-10A.

### Sphere formation assays

For the determination of sphere forming activity, adherently growing NSCLC cell lines H1299 and A549 were harvested and re-suspended in sphere-forming medium, containing 0.4% bovine serum albumin (Sigma-Aldrich), 10 ng/ml Epidermal Growth Factor (Sigma-Aldrich), 20 ng/ml Basic Fibroblast Growth Factor (VWR, Søborg, Denmark) and, in the case of H1299, 0.25 μg/ml human insulin (Sigma-Aldrich). To ensure a single cell suspension, the cells were filtered through 20 μm Steriflip filters (Millipore, Hellerup, Denmark), before being counted on a Cedex XS cell counter (Roche). The cells were seeded at densities of 250, 500, 1000, 2000, 4000 or 8000 cells/well in 150 μl medium in Corning^™^ Ultra-Low Attachment 96-Well Plates (Sigma-Aldrich). At day 6, spheres were counted under the microscope. Three pictures were taken per well, and the diameter of spheres was determined with the Cell^F program (Olympus Europe). For the sphere-based viability assay, 20μl CellTiterBlue^™^ (Promega, Nacka, Sweden) was added per well, and the plate incubated for 23 hours prior to viability readout with a Wallac Victor3 1420 Multilabel Counter (Perkin Elmer, Skovlunde, Denmark) at 560_ex_/590_em_ nm. Experiments were performed with 10 wells per setting, and repeated three times.

### Radiochemistry and subcellular distribution

DOTA-conjugated AS1411 (Trilink Biotechnologies, CA, USA) and DOTATATE (ABX, Radeberg, Germany) were labeled with ^57^Co. AS1411 was mixed with potassium acetate (0.1 M, pH 4.5) and ^57^CoCl_2_ (Perkin Elmer). The mixture was heated for 3 min at 850 W in a domestic microwave. The mixture was allowed to cool to room temperature, and potassium phosphate buffer 0.1 M, pH 7.55 was added. The aptamer solution was annealed for 1 min in a domestic microwave at 850 W. The radiochemical purity was analyzed using size exclusion HPLC employing a Biosep S2000 (Phenomenex, Værløse, Denmark) eluted with phosphate buffered saline. For labeling of DOTATATE, see [20]. H1299 cells were seeded as adherent cells and spheres in 24-well conventional and Ultra-Low Attachment plates, respectively using a uniform seeding density of 10,000 cells per well corresponding to 20,000 cells per ml. Three days after seeding, cells were incubated in serum-free medium containing the radioactive labeled conjugates, at a final concentration of 20 kBq/ml. The subcellular distribution of ^57^Co-DOTA-AS1411 (specific activity 49.4 MBq/nmol) and ^57^Co-DOTATATE (specific activity 4 MBq/nmol) was analyzed at the indicated time points. Non-labeled DOTA-AS1411 or DOTATATE in 1000-fold excess, were added to a separate set of wells for blocking of specific uptake (“block”) and evaluated at the late time points as previously described [20]. The radioactivity in the different cell fractions was measured in a 2470 Wizard Automatic Gamma Counter (Perkin Elmer). A separate series of cells was seeded in parallel and counted at the respective time points in order to normalize to cell number. The experiment was performed three times in triplicate.

### Statistical analyses

The Student’s t test was uniformly used to evaluate statistical significance. p-values < 0.05 were considered statistically significant.

## Results

### Expression of SSTR2 and NCL in cancer versus normal cells

**Fig 1:**
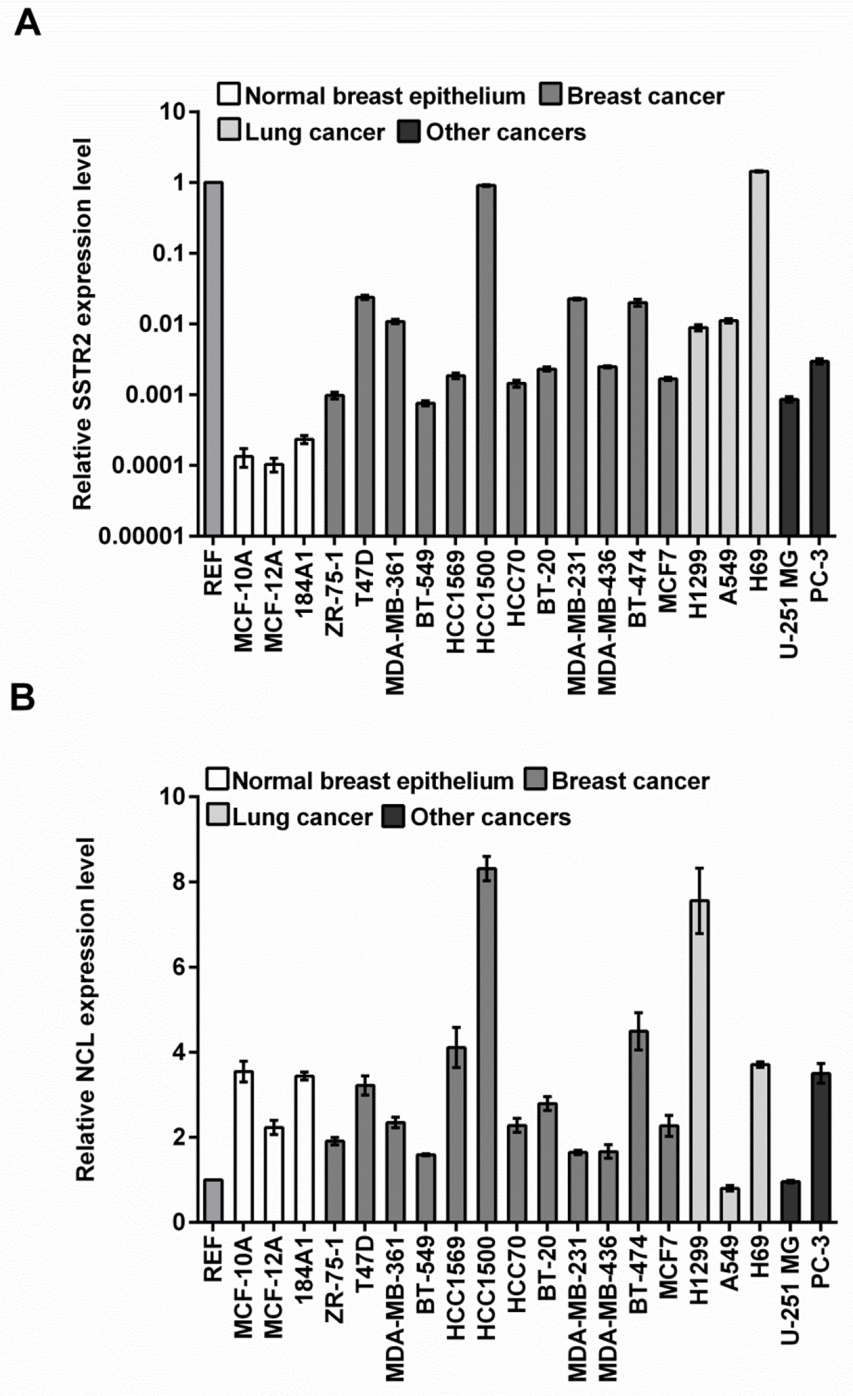
Real-Time PCR quantification of mRNA. Real-time PCR showing relative expression levels of *SSTR2* (A) and *NCL* (B) in a panel of human cell lines displayed on a logarithmic and a non-logarithmic scale, respectively. Expression levels were normalized to *GAPDH* and *ACTB* as housekeeping genes. We defined a common reference value (REF; see Materials and methods), to allow for comparison of expression levels between the two genes. The average level and SEM for each cell line were calculated from three wells.

In order to evaluate if SSTR2 and NCL can potentially be used for therapeutic delivery in NSCLC cells, we initially determined the relative mRNA expression levels by real-time PCR in the two NSCLC cell lines A549 and H1299 and in a panel of other cancer cell lines (Fig 1). For comparison, we used three normal, i.e. non-tumorigenic, mammary gland epithelial cell lines (MCF-10A, MCF-12A, and 184A1). *SSTR2* was higher expressed in all cancer cell lines versus the normal cell lines up to about 10,000-fold. The majority of the cancer cell lines displayed about 10- to 100-fold upregulation (56- and 70-fold respectively for H1299 and A549). However, the expression only caused elevation from a very low to a low or moderate level, since the majority of the cancer cell lines showed 100- to 1,000-fold reduced levels compared to the common reference value (Fig 1A). *NCL* showed a remarkably different pattern (Fig 1B). Only four cancer cell lines had elevated mRNA levels compared to normal epithelial cells, including the breast cancer cell line HCC1500 and the NSCLC cell line H1299 with about 2- to 3-fold increase. However, when comparing to the common reference value, *NCL* was in general expressed at substantially higher levels than *SSTR2*.

**Fig 2:**
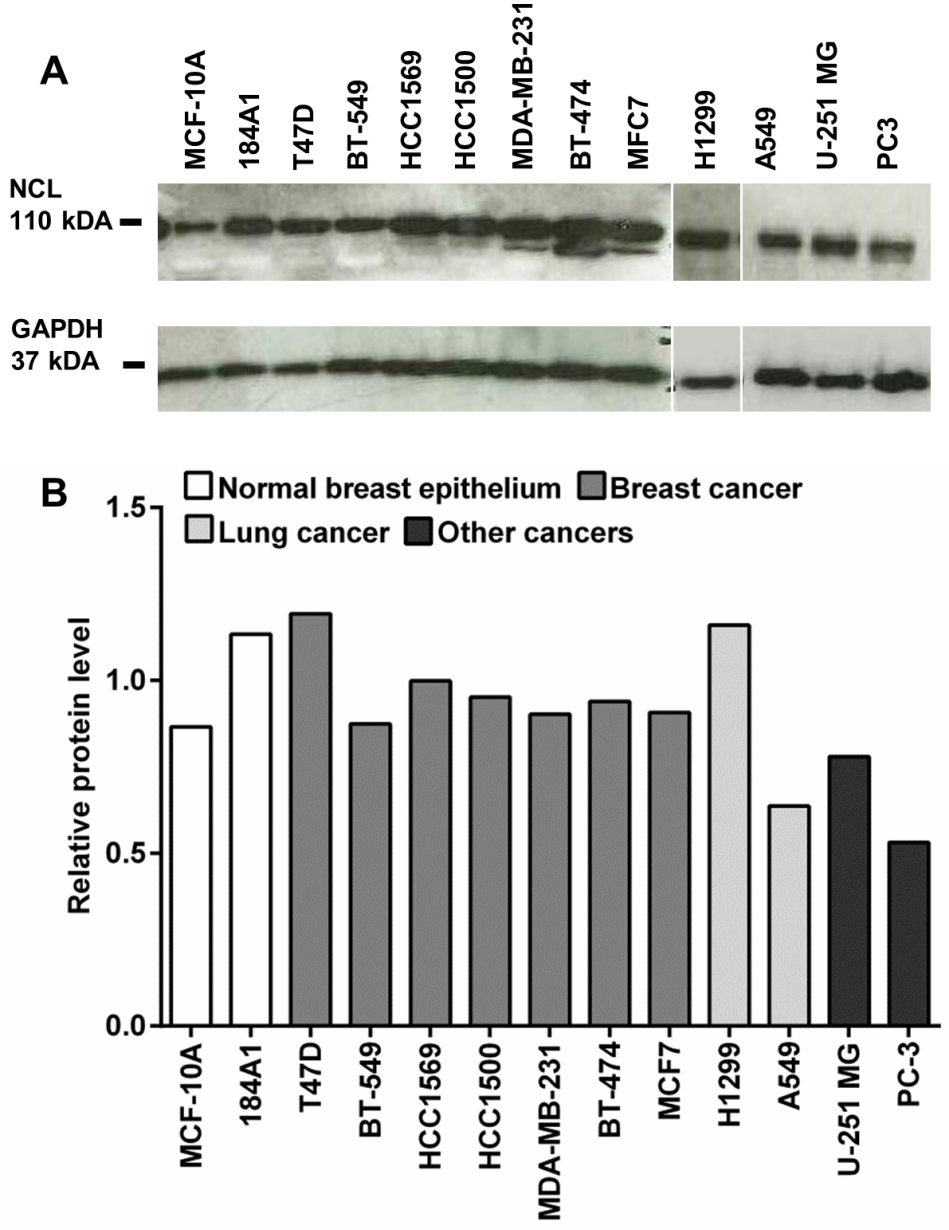
Western blot detection of NCL. (A) Western blot of extracts from different cell lines were first probed with anti-NCL antibody and afterwards reprobed with anti-GAPDH antibody for equal loading control. (B) Quantification of NCL expression relative to the expression of GAPDH and normalized to the mean value of the normal epithelial breast cell lines MCF-10A and 184A1.

Next, we evaluated the protein levels of NCL in a subset of the cell lines from the panel and quantified the expression relative to the expression levels of GAPDH (Fig 2). Albeit that protein levels seemed to be generally elevated in cancer versus normal cells, the increase was moderate.

These results suggest that *SSTR2* was selectively overexpressed in cancer cells, but at comparably low levels, while *NCL* was expressed at high overall levels but only displayed a moderate increase of protein levels compared to normal cells. Furthermore, both H1299 and A549 cells expressed about the same elevated levels of *SSTR2* compared to normal cells. In contrast, NCL was higher expressed in H1299 cells compared to A549 cells both at the mRNA and protein level.

### Differential sphere formation capacity

**Fig 3:**
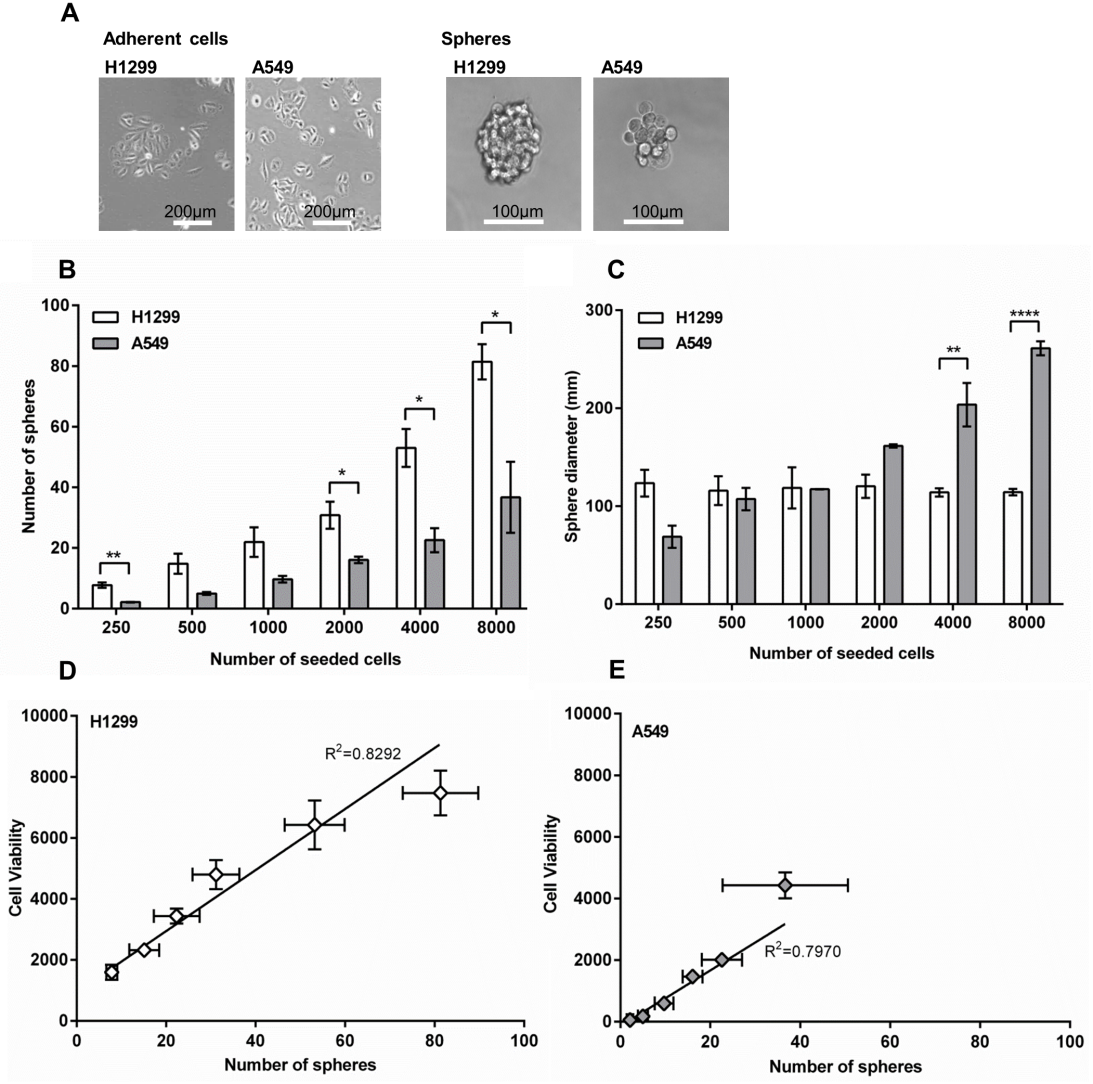
Sphere formation capacity. (A) The two NSCLC cell lines H1299 and A549 were grown as adherent cells or spheres for six days. (B) The number of lung cancer spheres after six days at different number of seeded cells. Mean and SEM from 3–4 plates. (C) Diameter of A549 and H1299 lung cancer spheres. Mean and SEM of 3–4 plates. The number of H1299 spheres (D) and A549 spheres (E) correlated with cell viability. SEM for sphere count and cell viability from 3–4 plates plotted as vertical and horizontal error lines, respectively. T-test depicts difference between A549 and H1299 at identical number og seeded cells p-value <0.05; *, <0.01; **; <0.0001; ****.

To establish a suitable test system for *in vitro* targeting studies of CSCs, we compared the sphere-forming abilities of A549 and H1299 cells. In non-adherent, serum-free culture conditions, non-CSCs undergo cell death by anoikis but CSCs survive to give rise to tumor spheres [21] so that, conceptually, the number of spheres is equivalent to the number of CSCs in the sample. In terms of general morphology, A549 cells formed poorly connected spheres, while H1299 cells formed more defined spheres (Fig 3A). Determination of sphere numbers at different seeding densities primarily suggested a good correlation for both NSCLC cell lines (Fig 3B). However, H1299 cells were about twice as potent as A549 cells in sphere formation. Regardless of seeding density, H1299 cells formed spheres of a uniform size of about 120 μm. By contrast, the size of A549 spheres increased from about 70 μm at the lowest seeding density to about 250 μm at the highest seeding density (Fig 3C). This strongly indicates that cell aggregation contributes to, or is responsible for A549 spheres, in agreement with the morphological appearance. Cell viability assays, used as alternative readout for spheroid formation, displayed good correlation with sphere numbers for both cell lines (Fig 3D and 3E). Overall, the sphere-forming ability of H1299 cells was significantly higher than the one of A549 cells, in which cell aggregation may add confounding effects. These results indicated H1299 cells as suitable model system for testing delivery by SSTR2 and NCL targeting.

### The NCL-targeting AS1411 aptamer shows efficient uptake by NSCLC CSCs

**Fig 4:**
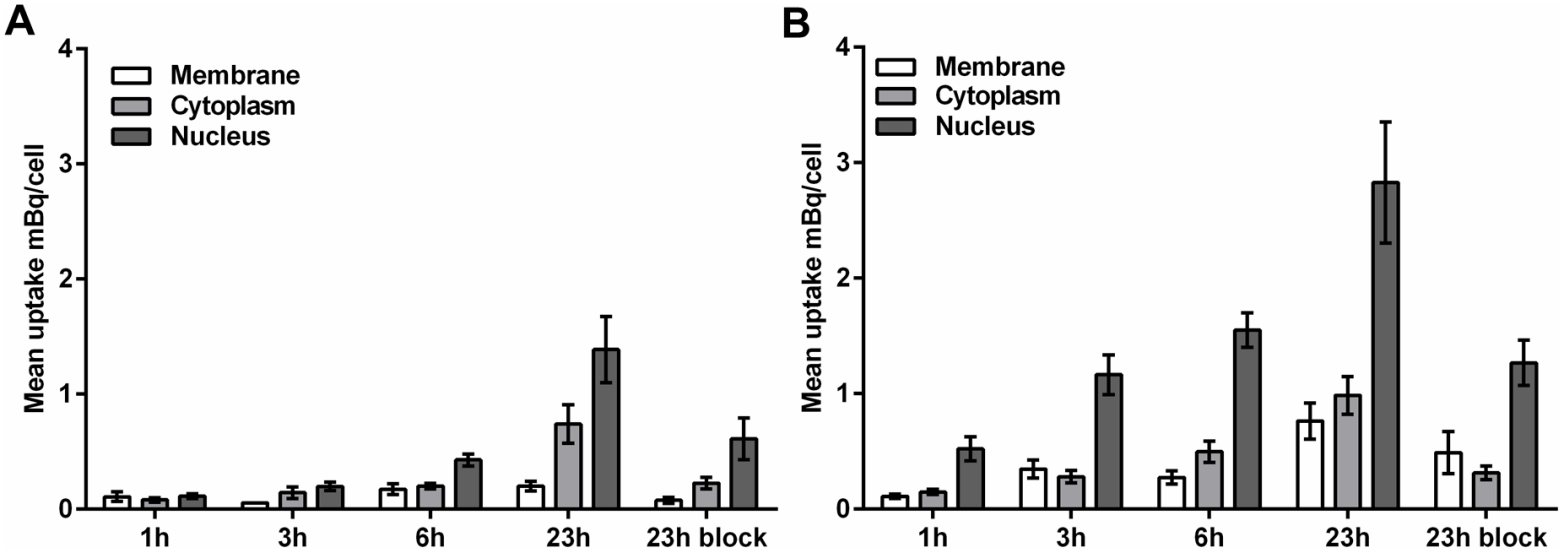
Uptake of ^57^Co labeled DOTATATE in the NSCLC cell line H1299. The subcellular distribution of ^57^Co-DOTA-AS1411 in H1299 adherent cells (A) and in H1299 spheres (B) as a function of increasing incubation time (1–23 h). In each case a blocking experiment was performed with 1000-fold excess AS1411 (23h block). Mean uptake (mBq/cell) is expressed as mean ± SEM from triplicate experiments.

Next, we explored SSTR2 and NCL targeting for potential delivery to NSCLC-CSCs, by performing uptake studies with radioactive labeled SSTR2-targeting peptide (^57^Co-DOTATATE) and NCL-targeting AS1411 aptamer (^57^Co-DOTA-AS1411) in both H1299 adherent cells and spheres. In agreement with the low expression levels of SSTR2, there was no uptake of ^57^Co-DOTATATE in H1299 non-CSCs or CSCs. In contrast, there was a time-dependent uptake of ^57^Co-DOTA-AS1411 in both H1299 non-CSCs (Fig 4A) and CSCs (Fig 4B). Interestingly, the total cell-associated ^57^Co-DOTA-AS1411 uptake was higher in CSCs compared to non-CSCs at all time-points. Most of the radioactivity was found in the nuclear fraction in both cell-types and up until the latest time point investigated (23 hours), uptake of ^57^Co-DOTA-AS1411 continued to increase, providing an about 2-fold increase in both total and nuclear uptake in CSCs versus non-CSCs (Fig 4A and 4B). The competitive blocking experiment indicated that most of the uptake was specific, although internalization was not completely blocked. The results show that the NCL-targeting AS1411 was efficiently internalized by non-CSCs and even more efficiently by CSCs from NSCLC and could be used for therapeutic delivery.

## Discussion

Evidence has accumulated that NSCLC, as suggested for other solid cancer types, is driven by CSCs [6, 7]. One particular hallmark of CSCs is drug resistance, caused by rapid extrusion of chemotherapeutics and other small molecule drugs, via multidrug resistance transporter systems such as ABCG2, which has been found on CD133-positive putative NSCLC-CSCs [6]. Opposed to this, macromolecular drugs, such as nucleic acids causing RNA-interference, and peptides would not be subjected to these drug resistance mechanisms [8–11, 22]. However, especially nucleic acids bear the problem of efficient delivery to cancer cells [9–11]. We here evaluated two broadly discussed cell surface targets, namely SSTR2 and NCL, for their ability to confer uptake in NSCLC-CSCs *in vitro*.

Both SSTR2 and NCL have previously been reported to be overexpressed by cancer cells [13–17]. Our present data confirms that SSTR2 is upregulated in cancer versus normal cell lines by a factor of 10- to 10,000-fold, however the SSTR2 expression levels in cancer cells were very low compared to the reference, also in the two NSCLC cell lines (Fig 1). By contrast, most cancer cell lines displayed NCL expression levels lower or equal to those observed in normal epithelial cell lines, and a moderate 2- to 3-fold increase in a smaller subset of the cancer cells at the mRNA level. These findings are largely recapitulated at the protein level, albeit NCL levels were equal to or higher in cancer cells versus normal epithelial cells. However, with about 2-fold, the increase was moderate (Fig 2). For yet unknown reasons, NCL is also present at the cell surface [17]. As the western blot analysis reflects the total protein, we cannot rule out that cancer cells may expose more dramatically elevated NCL levels at the surface than normal cells as has been found in other studies [23–25]. On the other hand, our uptake studies indicate that cell surface NCL is trafficked to the nucleus, which represents the predominant localization of the protein.

While the data argued that SSTR2 opposed to NCL would represent a cancer-specific molecule for targeting, we also included a common reference to gain an estimate of the overall intensity of expression of *SSTR2* and *NCL*. This methodology neither takes into consideration that the qRT-PCR assays for *SSTR2* and *NCL* may have different performance, nor the different levels of post-transcriptional and -translational regulation. Thus, differences are not necessarily expected to reflect true numerical differences. However, they may point to the correct order of magnitude.

Different markers and their combinations are currently discussed for NSCLC-CSCs [6, 7]. Expanding on the characterization of NSCLC-CSC markers was not the focus of our present study. Instead, in order to identify a suitable test system for uptake by NSCLC-CSCs, we approached the problem phenomenologically, by utilizing a marker-independent sphere formation assay. H1299 cells formed spheres more efficiently than A549 cells, for which cell aggregation may partly contribute to the rather irregularly shaped spheres. This data is in accordance with recent reports, indicating decreased sphere formation potential and *in vivo* tumorigenicity of A549 versus H1299 cells [26] and in turn may suggest a higher CSC content for H1299 than for A549 cells (Fig 3B and 3C). To this end, we identified it as suitable and robust test system for studying uptake in NSCLC-CSCs. We verified that the cells were CSCs by performing qPCR assays to analyze the expression of the three known markers oct4, sox2 and nanog (S1 Fig). All three markers were upregulated in cancer stem cells compared to bulk cancer cells supporting the notion that the cells grow under sphere conditions were truly CSCs.

Utilizing this model system, there was no uptake of ^57^Co-DOTATATE by H1299 cells. This was also supported by the mRNA analysis, which showed low levels of SSTR2 expression in the NSCLC cell lines. ^57^Co-DOTA-AS1411 on the other hand displayed efficient uptake and 4.5-fold higher in CSCs versus non-CSCs after 3 hours. At later time points, CSCs internalized about 2-fold the amount of the ^57^Co-DOTA-AS1411 internalized by non-CSCs. These results are in line with a recent study, in which Fonseca *et al*. reported efficient internalization of NCL-targeting liposomes by both breast cancer non-CSCs and CSCs [27]. Fonseca *et al*. associated increased *NCL* mRNA levels in stem cells with improved uptake, but increased accessibility or faster trafficking in CSCs could also represent conceivable reasons. We also cannot rule out that the absence of serum from the medium used for sphere cultivation contributes to the increased uptake by CSCs, albeit 0.4% bovine serum albumin was contained. It was recently reported that prolonged exposure *in vitro* to AS1411, causes a switch from NCL-dependent internalization to an NCL-independent macropinocytosis, which is triggered by the aptamer itself [28]. The competitive blocking experiments indicated that at 23 h, the NCL-dependent uptake still dominated (Fig 4A and 4B). Albeit, AS1411 previously has been shown the have anti-cancer activity in malignant cells, we saw no effect on proliferation and stemness (S2 Fig). This is most likely due owing to the fact that we only use 0.2 nM AS1411 (corresponding to 20 kBq/ml ^57^Co-AS1411), which is far below the 1–10 uM GI50 (50% growth inhibition) found for most malignant cell lines [29].

In conclusion, our data indicate that NCL-targeting via AS1411 in principle can be utilized for efficient delivery of radionuclides to NSCLC-CSCs. This might be accompanied by a trade-off that is to be made between selectivity for cancer cells and efficient uptake.

## Supporting information

S1 Fig mRNA expression of CSC markers in H1299 spheres and bulk cancer cells. The expression levels of oct4, sox2 and nanog were normalized to actin. Data and error bars are presented as mean ± SEM. ***p<0.001, ****p<0.001. The experiments were repeated three times.
(TIF)

S2 Fig Effect of AS1411 on proliferation and stemness. H1299 spheres and bulk cells were incubated with 0.2 nM AS1411 for 24h. (A) Proliferation was measured by the WST assay. Relative expression levels of oct4, sox2 and nanog were analyzed by qPCR in spheres (A) or in bulk cancer cells (B). Expression levels were normalized to actin and control cells (no AS1411). Data and error bars are presented as mean ± SEM. All the experiments were repeated three times.
(TIF)

S1 File Supplementary methods. (DOCX)
