# Supplementary material for: Evaluation of somatostatin and nucleolin receptors for therapeutic delivery in non-small cell lung cancer stem cells applying the somatostatin-analog DOTATATE and the nucleolin-targeting aptamer AS1411
Source: PLoS One. 2017 May 22;12(5):e0178286. doi: 10.1371/journal.pone.0178286 (PMC5440050; doi:10.1371/journal.pone.0178286)
Supplement: S1 File — (DOCX) [file pone.0178286.s003.docx]

**Supplementary methods**

**qPCR for stemness markers**

RNA was extracted from adherent or spheroid H1299 cells as described in materials and methods. qPCR was performed as described using the following Taqman Assays (all from Life Technologies) for *oct4* (*pou5f1*) (Hs00999632_g1), *sox2* (Hs01053049_s1) and *nanog* (Hs0460366_g1).

**Proliferation assay**

H1299 cells were grown as spheres and adherent cells in 96 well plates and incubated with 0.2 nM AS1411 for 24h. For measuring the proliferation, 15µl WST-1 (Roche) was added to each well. Absorbance was measured at 450/630 nm in an Absorbance Micoplate reader (ELX800, Biotek).

# Cell culture

Cell lines were grown in a humidified incubator (at 37 °C and 5% CO_2_). For passaging, cells were washed with phosphate buffered saline, and detached with TrypLE Express (Invitrogen, Karlsruhe, Germany). A549 (ATCC, CLL-185) was grown in Dulbecco’s Modified Eagle’s Medium (Sigma-Aldrich, Copenhagen, Denmark), and H1299 (ATCC, CLR-5803) was grown in Roswell Park Memorial Institute 1640 medium with L-glutamine (Sigma-Aldrich). Both media were supplemented with 10% fetal bovine serum (Sigma-Aldrich), and 1% Penicillin-Streptomycin (Sigma-Aldrich). All experiments were performed with 80% confluent cells. All pictures of cells were taken using the IX71® Inverted Microscope (Olympus Ballerup, Denmark), with a 10X/0.3phC lens.

In order to create the comparative RNA and protein panels, the following cell lines were used; 184A1 (CRL-8798), MCF-10A (CRL-10317), MCF-12A (CRL-10782), ZR-75-1 (CRL-1500), MCF7 (HTB-22), T47-D (HTB-133), MDA-MB-361 (HTB-27), BT-474 (HTB-20), HCC70 (CRL-2315), BT-20 (HTB-19), MDA-MB-231 (CRM-HTB-26), HCC1500 (CRL-2329), HCC1569 (CRL-2330), BT-549 (HTB-122), MDA-MB-436 (HBT-130), NCI-H69 (HTB-119), PC-3 (CRL-1435) and U-251 MG (formerly known as U-373 MG) (ECACC 09063001). All were obtained from ATCC (Wesel, Germany) except for U-251 MG that was obtained at the ECACC (Porton Down, UK). Cell lines were maintained following the guidelines of the providers.

# Quantitative RT-PCR

Total RNA was harvested from adherent cells using the RNeasy Mini Kit (Qiagen, Copenhagen, Denmark) according to the instructions of the supplier. The RNA concentration was measured on the NanoDrop ND-1000 spectrophotometer (Thermo Fisher Scientific, Hvidovre, Denmark), and stored at -80 °C until use. RNA was reverse transcribed using the RevertAid Minus First strand cDNA synthesis kit (Life Technologies, Nærum, Denmark) using 1 µg total RNA and oligo(dT) primers, following the instructions of the manufacturer. qRT-PCR was performed with Maxima® Probe/ROX qPCR Master Mix (Thermo Fisher Scientific) and taqman assays for *NCL* (Life Technologies, Hs01066668-m1) and *SSTR2* (Life Technologies, Hs00265624_s1). 10 ng cDNA was used per reaction, as recommended by the providers’ protocols. *ACTB* (Life Technologies, Hs99999903-m1) and *GAPDH* (Life Technologies, Hs99999905-m1) were used as references for normalization. The qRT-PCR was performed in a ABI Prism 7300 (Thermo Fisher Scientific), for 2 min at 50 °C, followed by 15 min at 95°C and 40 cycles at 95 °C for 15 sec and 60 °C for 1 min. Expression levels were analyzed using the Biogazelle qBase^plus^ software (www.qbaseplus.com), and normalized to *ACTB* and *GAPDH*. Moreover, the expression levels were referred to a virtual common reference (REF), representing the mean expression level of the *TP53* and the *CDKN1A* mRNA in MCF7 cells.
